# Natural grasslands across mainland France: A dataset including a 10 m raster and ground reference points

**DOI:** 10.1016/j.dib.2023.109348

**Published:** 2023-06-27

**Authors:** Léa Panhelleux, Sébastien Rapinel, Laurence Hubert-Moy

**Affiliations:** University of Rennes 2, LETG UMR 6554 CNRS, place du Recteur Henri Le Moal, 35000 Rennes, France

**Keywords:** Agriculture, Biodiversity, Field data, Green corridors, LULC, Meadows, Natural habitats, Sentinel-2

## Abstract

The data provided here include the first 10 m raster of natural grasslands across mainland France and related ground reference points. The latter consist of 1770 field observations that describe natural and artificial grasslands from respectively a compilation of hundreds of field-based vegetation maps and the European Union Land Parcel Identification System (LPIS). Based on analysis of aerial images, ground reference points were manually extracted from grassland polygons of the field-based vegetation maps and the LPIS within herbaceous areas larger than 30 × 30 m. The raster data of natural grasslands were derived from five annual 10 m land cover maps of France from 2016−2020. Pixels classified as ``grassland'' every year from 2016−2020 were considered natural grasslands, while those classified as ``crop'' at least once were considered artificial grasslands. Validation using the ground reference points revealed that natural and artificial grasslands were accurately mapped (overall accuracy = 86%). The ground reference points, publicly available in GeoJSON vector format, can be used as training or test samples for spatial modeling. The natural grassland map, publicly available in GeoTIFF raster format, can be used as a predictor variable for spatial modeling or as a base map for landscape ecology analyses.

**Specification Table**

**Table:** 

Subject	Nature and Landscape Conservation
Specific subject area	GIS, natural habitat, remote sensing
Type of data	GIS raster and vector
How the data were acquired	The 1770 reference points for natural and artificial grasslands were respectively derived from vegetation maps and the Land Parcel Identification System (LPIS). The 10 m raster of natural grasslands was derived from five annual land cover maps from 2016−2020. All data were produced in a geographical information system using QGIS software [1].
Data format	Analyzed (GIS raster in GeoTIFF format, and vector in GeoJSON format)
Description of data collection	The reference points were collected in 4 steps: • Selection of natural and artificial grassland polygons using vegetation maps and LPIS respectively, • Spatial subsampling using 20 × 20 km grids, • Exclusion of polygons < 1 ha, • Visual extraction of one reference point per grassland polygon within an herbaceous area larger than 30 × 30 m (excluding e.g. bare soils and isolated trees) using aerial images. The raster of natural grasslands was created in 2 steps: • Calculation of grassland frequency per pixel from 2016-2020, • Classification of natural grasslands (i.e. pixels classified as grasslands every year).
Data source location	Mainland France The raw data used to derive the dataset were provided by regional offices of the environment for the field-based vegetation maps, the French National Geographic Institute (IGN) for the LPIS and aerial images, and the French national land surface data and services hub (Pôle Theia) for the land cover maps.
Data accessibility	Secondary data The dataset of natural grasslands is publicly provided as follows: Repository name: Zenodo Data identification number: 10.5281/zenodo.7895449 Direct URL to data: https://doi.org/10.5281/zenodo.7895449 Primary data The LPIS is released in vector format as follows: Repository name: Geoservices (IGN) Data identification number: IGNF_RPG_2-0 Direct URL to data: https://geoservices.ign.fr/rpg#telechargementrpg2020 Aerial images are released in raster format by French department as follows: Repository name: Geoservices (IGN) Data identification number: IGNF_BDORTHOr_2-0 Direct URL to data: https://geoservices.ign.fr/bdortho The 2016 land cover map is released in raster format as follows: Repository name: Pôle thématique Surfaces Continentales Theia (CNES) Data identification number: OSO_20160101_RASTER Direct URL to data: https://theia.cnes.fr/atdistrib/rocket/#/collections/OSO/885099e0-aac5-5184-9345-e1b62a55f2ab The 2017 land cover map is released in raster format as follows: Repository name: Pôle thématique Surfaces Continentales Theia (CNES) Data identification number: OSO_20170101_RASTER Direct URL to data: https://theia.cnes.fr/atdistrib/rocket/#/collections/OSO/56364a89-2546-57f3-810b-41deef11e176
	The 2018 land cover map is released in raster format as follows: Repository name: Pôle thématique Surfaces Continentales Theia (CNES) Data identification number: OSO_20180101_RASTER Direct URL to data: https://theia.cnes.fr/atdistrib/rocket/#/collections/OSO/c2032fa2-95f3-5dff-866f-b3e2fe5af7d8 The 2019 land cover map is released in raster format as follows: Repository name: Pôle thématique Surfaces Continentales Theia (CNES) Data identification number: OSO_20190101_RASTER Direct URL to data: https://theia.cnes.fr/atdistrib/rocket/#/collections/OSO/12e01f87-4e6d-5788-8063-ce59918dae14 The 2020 land cover map is released in raster format as follows: Repository name: Pôle thématique Surfaces Continentales Theia (CNES) Data identification number: OSO_20200101_RASTER Direct URL to data: https://theia.cnes.fr/atdistrib/rocket/#/collections/OSO/2327b748-a82c-5933-afb0-087bbfeff4cd

## Value of the Data

• The ground reference points, which accurately geolocate the presence of natural grasslands, were obtained by compiling hundreds of field-based vegetation maps across France.
• The 10 m raster of natural grasslands, based on five years of monitoring, is the first map of such habitats available at high spatial resolution for the whole mainland of France.
• These data can benefit scientists and stakeholders in the fields of ecology, agriculture, or land-use planning.
• The ground reference points can be used as training or test samples for spatial modeling.
• The natural grassland map can be used as a predictor variable for spatial modeling or as a base map in landscape ecology.

## 1 Objective

Ground reference points for natural grasslands across France were created since no open-source national database was available that compiled hundreds of field-based vegetation maps. A natural grassland map was created because current land cover products at high spatial resolution based on annual satellite time-series do not distinguish natural grasslands from artificial grasslands. The ground reference points were used to validate the accuracy of the 10 m raster of natural grasslands. The map of natural grasslands will be used as a base map to characterize the current fragmentation of grassland habitats in landscapes throughout France. This data paper describes the structure of these data and the method used to generate them for other users and uses.

## 2 Data Description

**Fig. 1:**
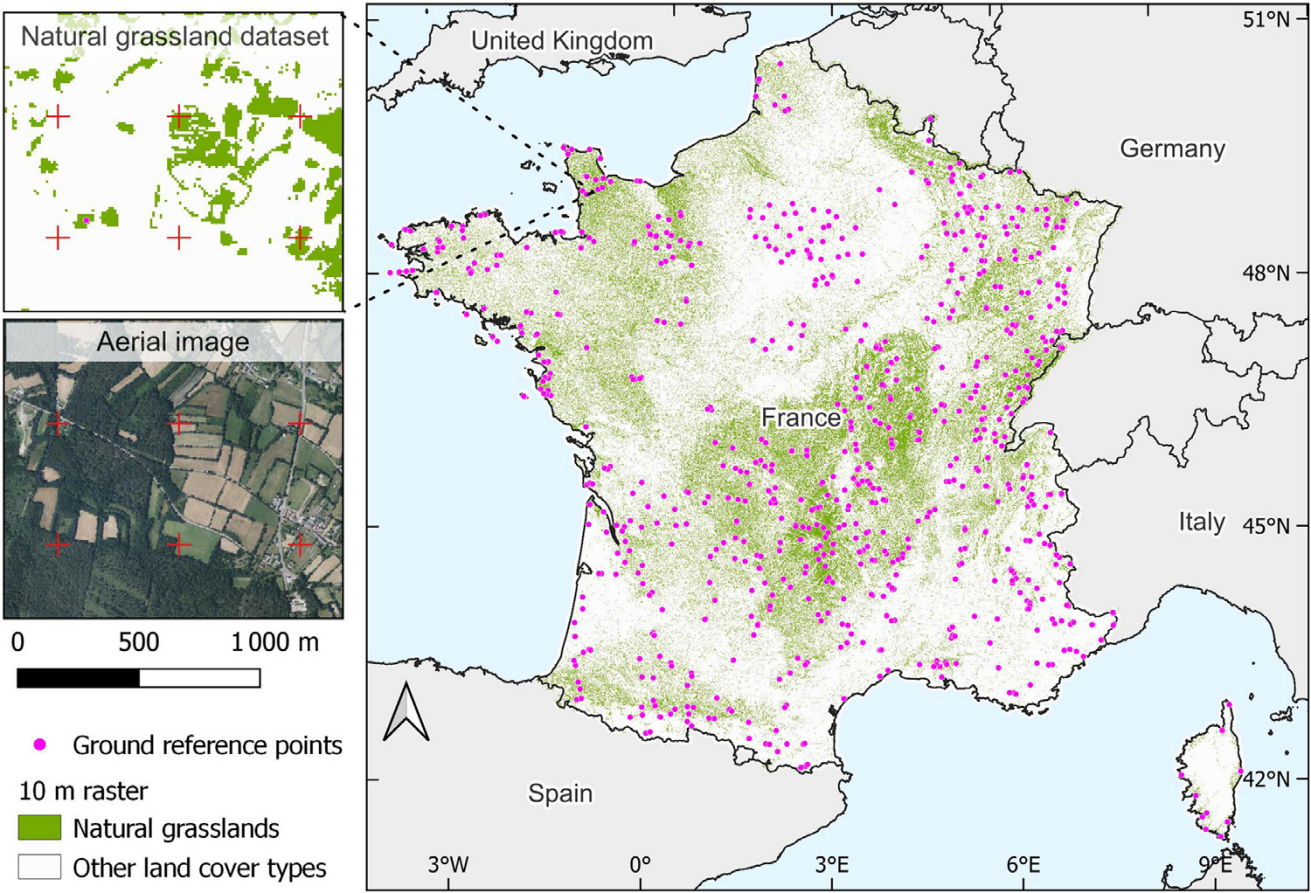
Ground reference points and pixels of the 10 m raster of the natural grassland dataset across mainland France. For clarity, those of artificial grasslands are not shown.

The dataset covers for the whole mainland of France (Fig. 1). The ground reference points are provided in standard open-source geospatial data interchange format GeoJSON (grassland_ground_points.geojson). These data are projected in the French Lambert-93 projection system (EPSG code 2154) and consist of 882 points for natural grasslands and 888 points for artificial grasslands (total: 1770). Each point is the centroid of a 30 × 30 m square of herbaceous area, excluding isolated trees, rivers, and impervious areas. The attribute table has two fields: ``ID'' (the identifier) and ``type'' (the type of grassland: ``natural'' or ``artificial''). A supplementary layout file (grassland_ground_points.qlr) supports formatting of the points in QGIS software.

The map of natural grasslands in 2020 is provided as a file in GeoTIFF format (natural_grasslands_2020.tif). It is a categorical raster at 10 m spatial resolution projected in the French Lambert-93 system covering all of mainland France. It includes natural grassland (code 1), but also artificial grassland (code 2) and other land cover types (code 3). A layout file (natural_grasslands_2020.qml) supports formatting of the map in QGIS software.

## 3 Experimental Design, Materials and Methods

Ground reference points for natural and artificial grasslands were extracted from field vegetation maps and the European Union (EU) LPIS, respectively. For natural grasslands, no national open-source database that included all field-based vegetation maps of France was available. Thus, local vegetation maps were requested from each of France's 13 regional environmental agencies. These vegetation maps cover mainly protected areas [2]. All vegetation maps were then combined in a GIS, and the following polygons, corresponding to natural grasslands, were selected using SQL queries: ``Grasslands and lands dominated by forbs, mosses or lichens'' (code E and lower nested levels) in the EUNIS classification system [3], ``Natural and semi-natural grassland formations'' (code 6 and lower nested levels) in the EU Habitat Directive Annex 1 classification system [4], and ``Humid grasslands and tall herb communities'' or ``Mesophile grasslands'' (code 37 and 38, respectively, and their lower nested levels) in the CORINE Biotopes classification system [5]. For artificial grasslands, the LPIS for 2020 was used. The LPIS is a polygon vector file that describes the crop type per field based on farmers' declarations [6]. Polygons classified as ``Temporary grassland less than 5 years old'' (code 19) were selected.

**Fig. 2:**
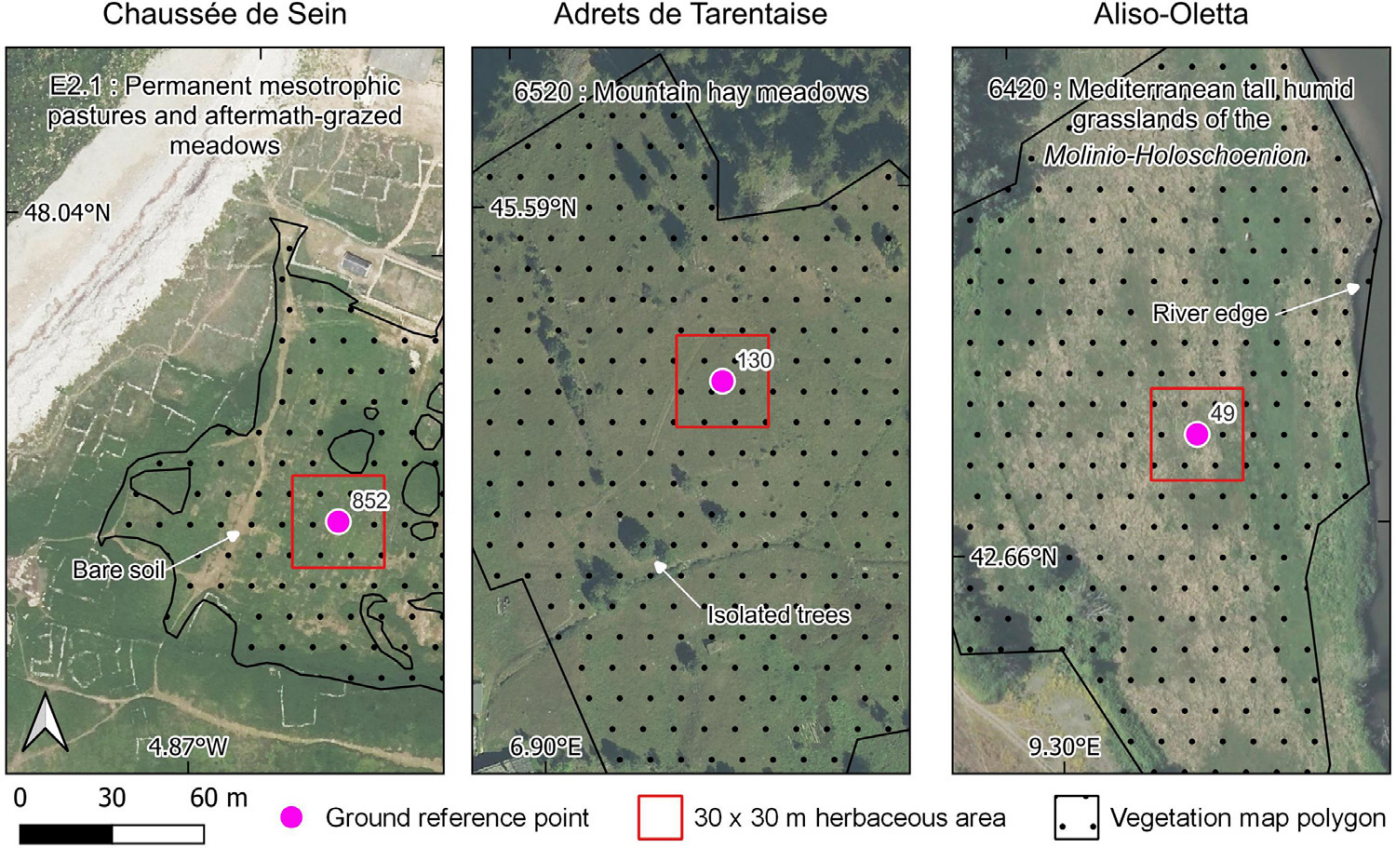
Examples of selection of natural grassland reference points in three Natura 2000 protected sites: (left) Chaussée de Sein (site code: FR5302007) in the Atlantic biogeographical region, (middle) Adrets de la Tarentaise (site code: FR8201777) in the Alpine biogeographical region, and (right) Aliso-Oletta (site code: FR9400601) in the Mediterranean biogeographical region. The number to the right of each point indicates its identifier.

The polygons of the vegetation maps and LPIS were joined, and only those larger than 1 ha were retained. To balance the spatial distribution of the polygons, sub-sampling was performed in 20 × 20 km grids, with a maximum of 10 polygons per grid for natural grasslands, and a maximum of 1 polygon per grid for artificial grasslands. Additional random subsampling was then performed to obtain a reasonable number (∼1000 per grassland type) of polygons for visual analysis. Each selected polygon was then visually analyzed in the most recent aerial image, and a reference point was placed at the centroid of each homogeneous herbaceous area that covered at least 30 × 30 m (Fig. 2). This grid size was set to ensure that the point corresponded to a pure Sentinel-2 pixel (10 × 10 m) of grassland. Polygons that did not meet the requirement for minimum grassland area (e.g. changes in land cover, narrow patterns) were excluded. Ultimately, 882 and 888 points were collected for natural and artificial grasslands, respectively.

**Fig. 3:**
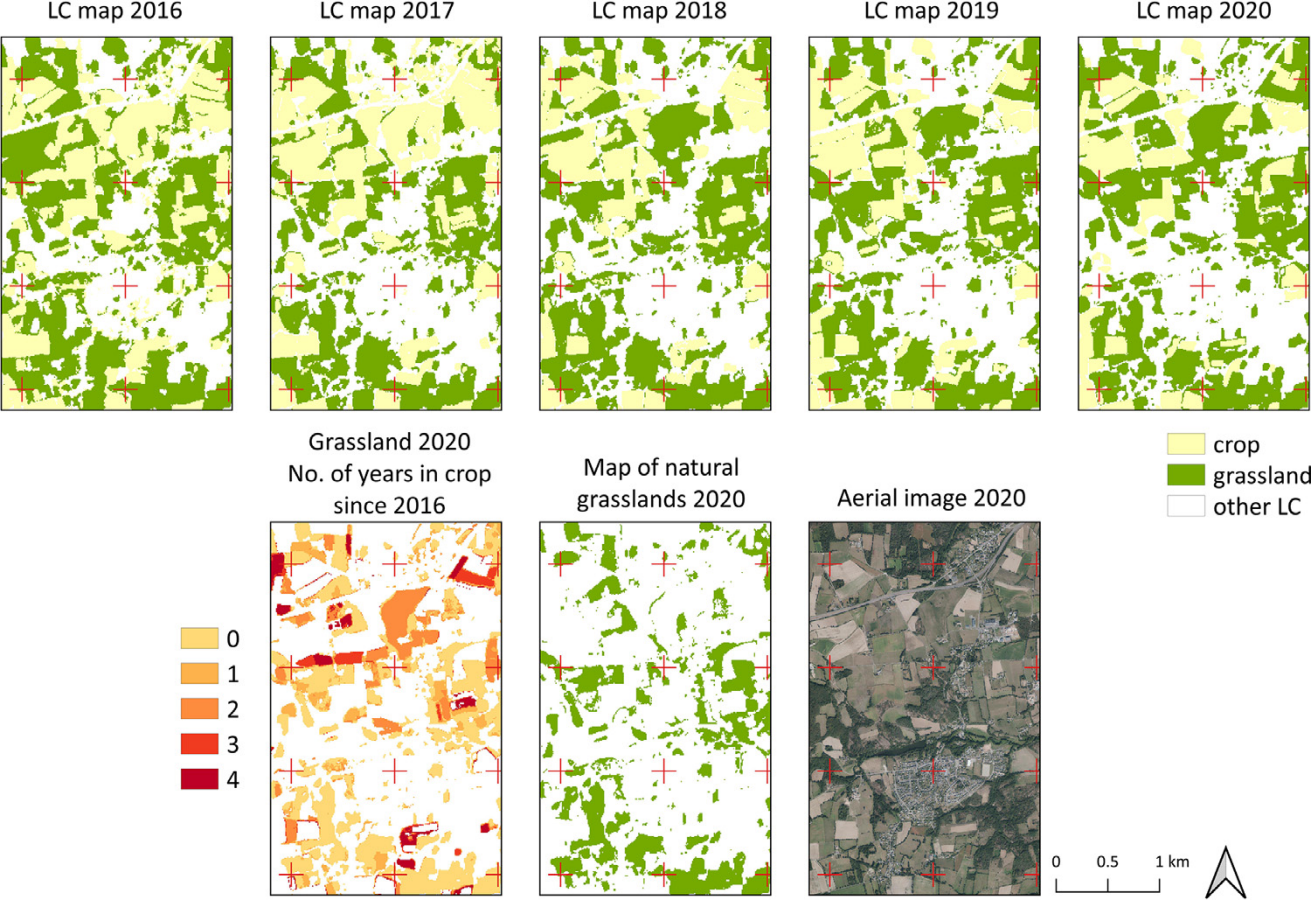
Illustration of the method used to generate the 10 m raster of natural grasslands in Brittany, France, in the Atlantic biogeographical region (48.03°N, 1.93°W): (top) the land cover (LC) maps from 2016–2020 were combined, (bottom) the number of years the 2020 grasslands had been cropped since 2016 was calculated, and the grasslands that had not been cropped were considered natural.

The 10 m raster of natural grasslands was derived from the five annual national land cover maps from 2016−2020 [7], [8], [9], [10], [11]. These land cover maps, which had been produced by automatically classifying Sentinel-2 time-series (overall accuracy = 88−91% [12]), were downloaded and stacked into a single raster file. Transition matrix analysis was performed to focus on grasslands (code 211 for 2016−2017, code 13 for 2018−2020) and crops (codes 11−12 for 2016−2017, codes 5−12 for 2018−2020). Pixels classified as grassland every year from 2016−2020 were considered natural grasslands (Fig. 3), while those classified as ``crop'' at least once were considered artificial grasslands. To support this reasoning, ground reference points were used to assess the accuracy of the classification of natural and artificial grasslands (Table 1). The overall accuracy was high (86%), especially since the few under-detection errors (user's accuracy) of natural grasslands were related mainly to confusion with non-grassland land cover types.

**Table 1:** Confusion matrix of the 10 m raster of natural grasslands (prediction) and the ground points (reference). ``Others'' refers to all non-grassland land cover types. NA = not applicable.

	Reference			
Prediction	Others	Artificial grassland	Natural grassland	Producer's accuracy
Others	0	5	98	0.00
Artificial grassland	0	799	69	0.92
Natural grassland	0	84	715	0.89
User's accuracy	NA	0.90	0.81	
				
F1-score	NA	0.91	0.85	

Overall accuracy: 0.86.

## CRediT authorship contribution statement

**Léa Panhelleux:** Formal analysis, Investigation, Software. **Sébastien Rapinel:** Methodology, Supervision, Writing – original draft. **Laurence Hubert-Moy:** Conceptualization, Methodology, Supervision, Project administration, Writing – original draft.

## Declaration of Competing Interest

The authors declare that they have no known competing financial interests or personal relationships that could have appeared to influence the work reported in this paper.

## Data Availability

Natural grasslands across mainland France: a dataset including a 10 m raster and ground reference points (Original data) (zenodo).
